# Thyroid Storm Presenting With Refractory Atrial Arrhythmia and Tachycardia-Induced Dilated Cardiomyopathy: A Case Report

**DOI:** 10.7759/cureus.114394

**Published:** 2026-08-12

**Authors:** Sweta Pillai, Favour O Balogun, Inithan Ganesaratnam

**Affiliations:** 1 Internal Medicine, Kingston Hospital NHS Foundation Trust, London, GBR; 2 Internal Medicine, James Paget University Hospital, Great Yarmouth, GBR; 3 Intensive Care Unit, Kingston Hospital NHS Foundation Trust, London, GBR

**Keywords:** antithyroid therapy, atrial flutter, dilated cardiomyopathy, graves disease, multidisciplinary management, refractory arrhythmia, tachycardia-induced cardiomyopathy, thyroid storm

## Abstract

Thyroid storm is a life-threatening endocrine emergency characterised by extreme hypermetabolic derangement. Cardiovascular complications, including supraventricular arrhythmias and high-output cardiac failure, are well-recognised; however, the combination of refractory atrial flutter, tachycardia-induced dilated cardiomyopathy and severe biventricular dysfunction represents an uncommon and particularly challenging presentation.

We report a 59-year-old woman with no prior cardiac history who presented with progressive malaise, exertional dyspnoea, palpitations and systemic symptoms. She was found to be in haemodynamically significant narrow complex tachycardia exceeding 200 beats per minute with a calculated Burch-Wartofsky score of 95, consistent with thyroid storm. Transthoracic echocardiography demonstrated severely impaired left ventricular systolic function with an ejection fraction of 15-20%, severe biatrial dilatation and echocardiographic evidence of pulmonary hypertension, consistent with tachycardia-induced dilated cardiomyopathy.

Multiple rhythm-control and rate-control strategies were required, including direct-current cardioversion, adenosine, magnesium, digoxin, beta-blockers and escalating infusions. Amiodarone was discontinued following multidisciplinary review due to concerns regarding iodine-induced exacerbation of thyrotoxicosis. Thyroid storm was treated with propylthiouracil, Lugol's iodine, hydrocortisone, propranolol and cholestyramine. Serial echocardiography demonstrated interval improvement in left ventricular systolic function, supporting a reversible tachycardia-mediated mechanism. This case highlights the diagnostic and therapeutic complexity of thyroid storm with refractory arrhythmia and underscores the importance of early recognition, multidisciplinary management and careful selection of antiarrhythmic therapy.

## Introduction

Thyroid storm is a rare but potentially fatal complication of severe thyrotoxicosis, with an incidence of approximately 0.2-1.1 per 100,000 in the general population and 4.8-6.3 per 100,000 among hospitalised patients, and is characterised by extreme physiological decompensation across multiple organ systems, with mortality rates of 5-25% despite modern critical care management [1]. In a large Japanese national inpatient database study of 1,324 patients with thyroid storm, the overall in-hospital mortality was 10.1%, with cardiovascular disease representing the most common comorbidity at admission (46.6%) [2].

The cardiovascular system is among the most profoundly affected organ systems, with thyroid hormone excess causing increased heart rate, augmented cardiac contractility, reduced systemic vascular resistance and heightened susceptibility to atrial arrhythmias [1]. In a national inpatient sample of 6,380 thyroid storm hospitalisations, arrhythmia was the most frequently associated cardiovascular event (N=3,770), followed by acute heart failure (N=555), with in-hospital mortality significantly higher in those with cardiovascular events compared to those without (3.5% vs 0.2%, P<0.005) [3].

Atrial fibrillation occurs in up to 15% of patients with hyperthyroidism compared to 4% of the general population, and is a well-recognised precipitant of haemodynamic instability [4]. Sustained tachyarrhythmia, when prolonged, may itself cause progressive ventricular dysfunction through tachycardia-induced cardiomyopathy - a potentially reversible form of dilated cardiomyopathy that can resolve with successful rate or rhythm control [5]. The coexistence of thyroid storm, refractory atrial arrhythmia and tachycardia-induced dilated cardiomyopathy presents a particularly complex management challenge, as treatment strategies for one condition may adversely affect another.

The pathophysiology underlying the transition from compensated thyrotoxicosis to thyroid storm remains incompletely understood, as the severity of biochemical hormone elevation alone does not predict which patients will decompensate. Although the clinical picture resembles catecholamine excess, circulating catecholamine levels are typically normal; increased tissue responsiveness and heightened β-adrenergic receptor activity are thought to underlie this hyperadrenergic phenotype, which also explains the clinical efficacy of beta-blockade. Precipitating factors are proposed to provoke rapid hormone surges or heightened tissue sensitivity via mechanisms including increased cytokine release and altered adrenergic receptor responsiveness, amplifying the hypermetabolic state. Cellular metabolic stress and uncoupling of oxidative phosphorylation, particularly during hypoxia or infection, may further increase thermogenesis and contribute to the systemic hyperthermia and multiorgan derangement characteristic of thyroid storm [1].

We present a case of thyroid storm in a 59-year-old woman with no prior cardiac history who developed haemodynamically significant atrial flutter refractory to multiple pharmacological and electrical interventions, complicated by severe biventricular dysfunction with echocardiographic features of pulmonary hypertension. This case illustrates the diagnostic complexity of such presentations and highlights the need for expedient multidisciplinary involvement in guiding therapeutic decision-making.

## Case presentation

A 59-year-old woman with no significant past medical history presented on Day 1 of admission with a two-week history of progressive malaise, exertional dyspnoea, palpitations and loose stools. Her only regular medication was semaglutide, prescribed for weight management. Collateral history from friends noted bilateral eye prominence and visible neck pulsations in the days preceding presentation.

**Figure 1:**
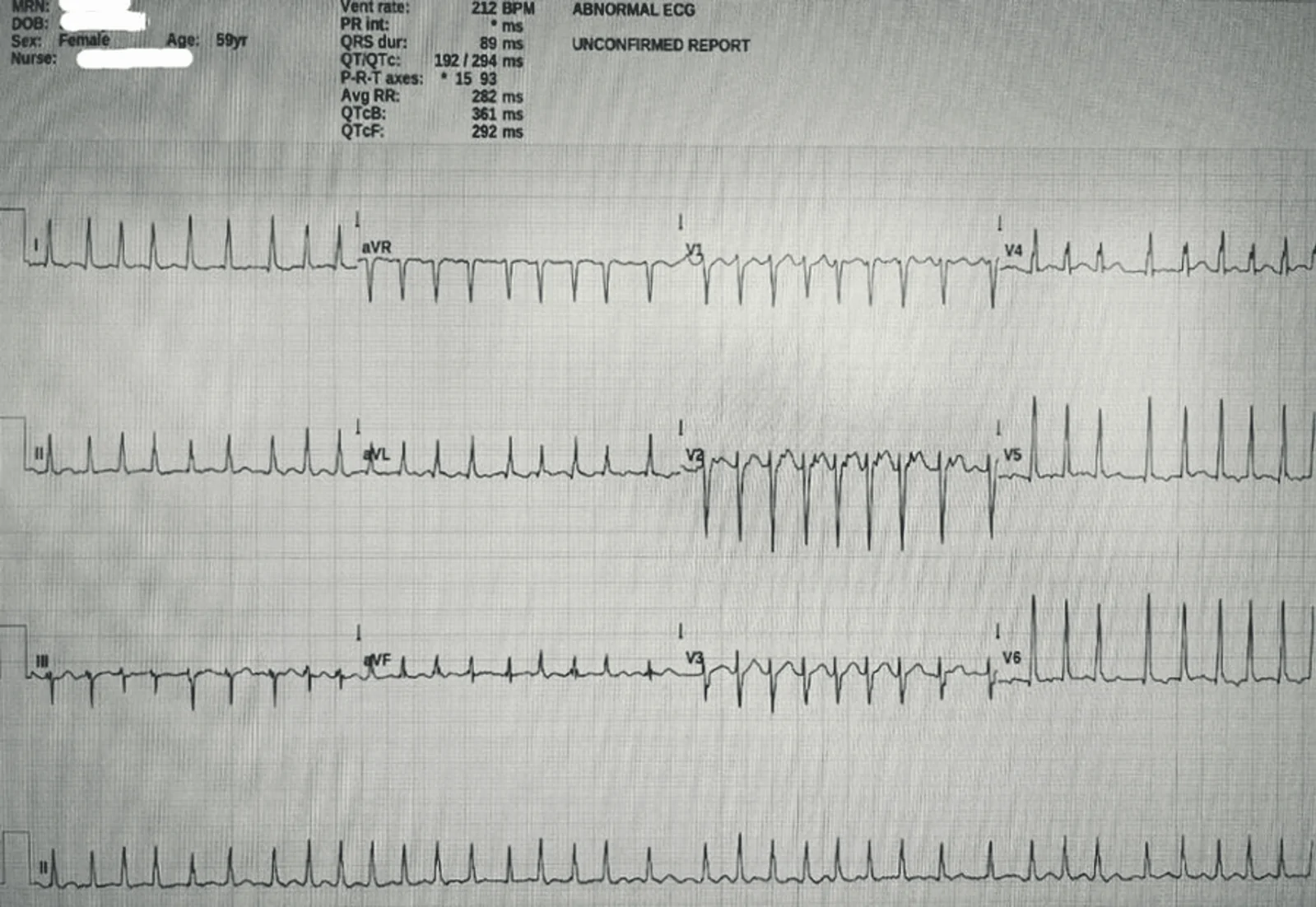
Twelve-lead electrocardiogram (ECG) at presentation demonstrating narrow-complex tachycardia at 212 beats/min, with a QRS duration of 89 ms, QTcB of 361 ms and QTcF of 292 ms, consistent with atrial flutter with variable block.

On initial assessment, she was haemodynamically unstable, with a heart rate exceeding 200 beats per minute. Clinical examination demonstrated bilateral proptosis, elevated jugular venous pulsations and pitting oedema to the thighs. Bilateral eye changes, combined with the systemic features, raised immediate clinical suspicion for Graves' disease with thyrotoxic decompensation [1]. A Burch-Wartofsky score of 95 was calculated using the original point scale for thyroid storm [6], which is highly suggestive of thyroid storm. A 12-lead ECG at presentation (Figure 1) demonstrated narrow complex tachycardia at 212 beats/min with a QRS duration of 89 ms, QTcB of 361 ms and QTcF of 292 ms, consistent with atrial flutter with variable block.

Initial laboratory investigations, summarised in Table 1, confirmed severe biochemical thyrotoxicosis, including a markedly elevated thyroid-stimulating hormone receptor antibody (TRAb) level, confirming an autoimmune aetiology consistent with Graves' disease. Inflammatory markers were not significantly elevated at presentation, and no clear infective source was identified. Chest imaging and further biochemical investigations were performed as part of the initial resuscitation workup.

**Table 1:** Key laboratory investigations with reference ranges. CRP: C-reactive protein; T3: triiodothyronine; T4: thyroxine; H: high; L: low; TSH: thyroid-stimulating hormone

Parameter	Patient Value	Reference Range
TSH receptor antibodies	34.71 IU/L (H)	0-0.39 IU/L
TSH	<0.01 mIU/L (L)	0.27-4.2 mIU/L
Free T4 (one month post-treatment)	32.9 pmol/L (H)	8-18 pmol/L
Free T3 (one month post-treatment)	11 pmol/L (H)	4.2-7.1 pmol/L
CRP (Day 2)	5.1 mg/L	0-5 mg/L
Troponin I (Day 2)	48 ng/L (H)	0-12 ng/L

Neck ultrasound

**Figure 2:**
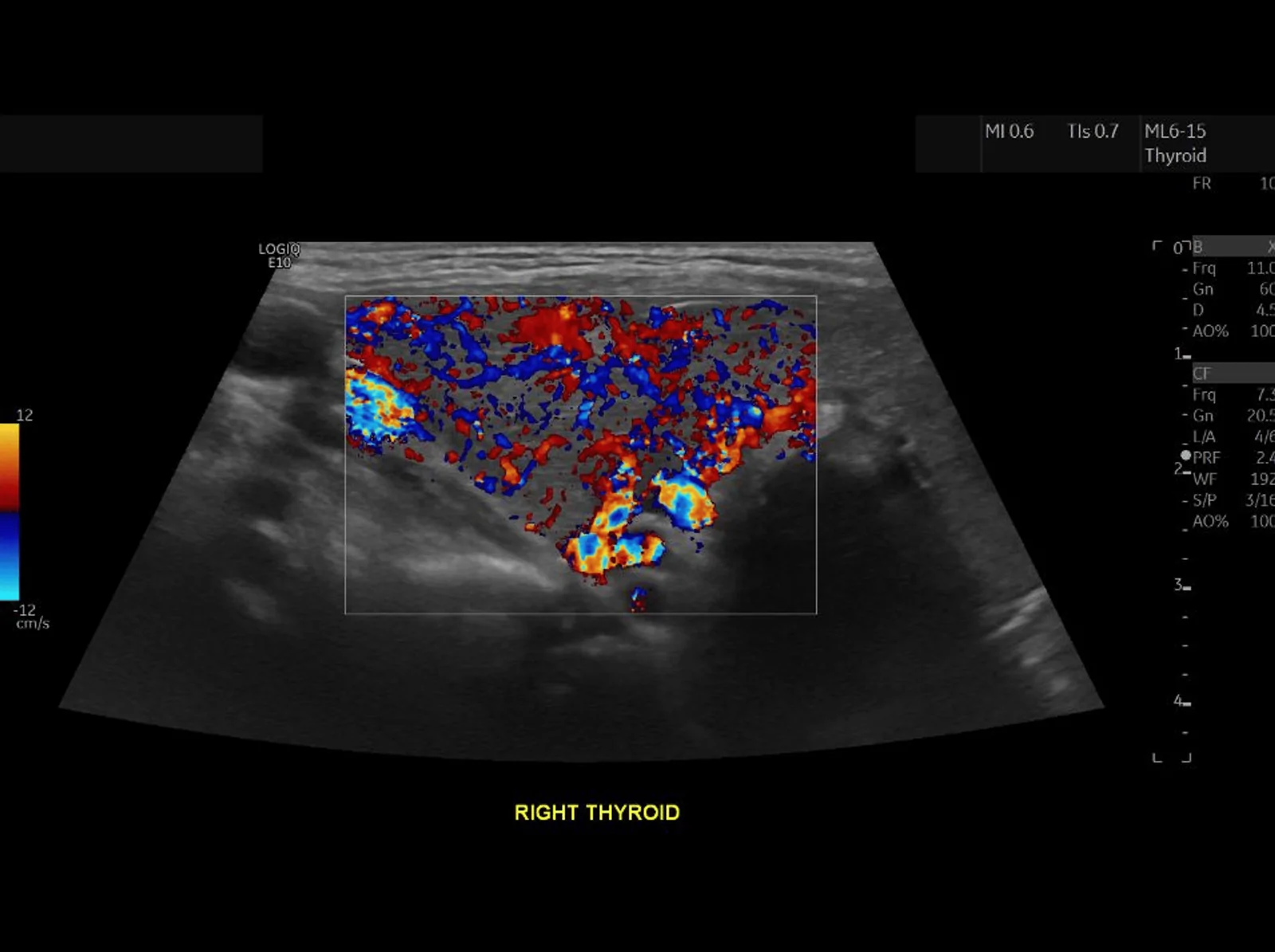
Color Doppler ultrasound of the right thyroid lobe demonstrating markedly increased vascularity, consistent with active thyroiditis.

Neck ultrasound (Figure 2) demonstrated generalised thyroid enlargement, with the right lobe measuring 40 x 20 x 27 mm and the left lobe 42 x 26 x 27 mm, without retrosternal extension or tracheal deviation. The parenchyma was hypoechoic and coarsened with markedly increased vascularity, in keeping with active thyroiditis. A 4 mm hyperechoic focus in the anterior right lobe likely represents preserved parenchyma or a benign (U2) nodule, and a 7 mm isoechoic nodule more posteriorly in the right lobe was classified as U2, requiring follow-up. No cervical adenopathy was identified. These appearances are consistent with Graves' disease. An endocrinology review was recommended to determine the need for nuclear medicine thyroid uptake scintigraphy, and a three-month ultrasound follow-up was advised.

Echocardiography

**Figure 3:**
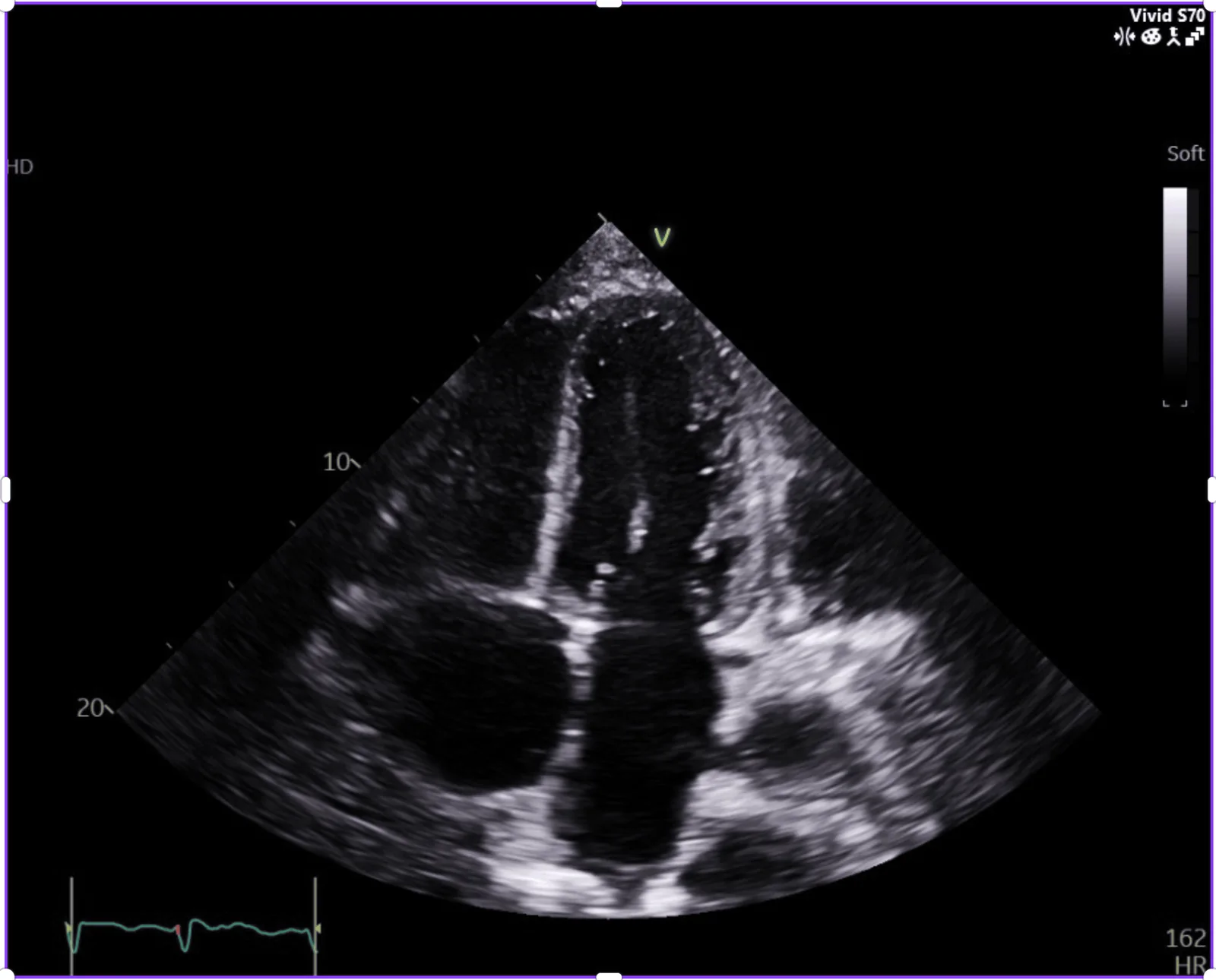
Apical four-chamber transthoracic echocardiogram at presentation, obtained during diastole phase, demonstrating a dilated left ventricle with severe biatrial dilatation.

Transthoracic echocardiography performed on Day 2, whilst the patient remained tachycardic at 160-170 beats per minute, is shown in Figure 3 and demonstrated a dilated left ventricle with severely impaired systolic function and an estimated ejection fraction of 15-20%, severe biatrial dilatation, moderate functional mitral regurgitation, and moderate-to-severe functional tricuspid regurgitation. The estimated right ventricular systolic pressure was 52 mmHg, indicating a high echocardiographic probability of pulmonary hypertension.

Repeat echocardiography on Day 5 demonstrated interval improvement in left ventricular systolic function with ejection fraction improving to approximately 30%, alongside improvement in functional tricuspid regurgitation and pulmonary hypertension parameters, supporting a reversible tachycardia-mediated mechanism [5,7].

Treatment

The patient was admitted to the intensive care unit and intubated due to worsening respiratory distress and persistent haemodynamically significant tachyarrhythmia. Vasopressor support with noradrenaline was temporarily required.

Rhythm and rate control

Initial rhythm-control strategies included three doses of intravenous adenosine (6 mg, 12 mg and 18 mg) and three direct-current cardioversions in the emergency department, all of which were unsuccessful. Subsequent rate-control measures included intravenous magnesium, digoxin loading, metoprolol boluses, labetalol infusion and esmolol infusion. An amiodarone infusion was commenced but subsequently discontinued following multidisciplinary review, due to concerns regarding worsening thyrotoxicosis secondary to its high iodine content and prolonged tissue accumulation [1]. A rate-control strategy was thereafter adopted, transitioning from propranolol to bisoprolol, titrated for ventricular rate control, alongside maintenance digoxin. Therapeutic anticoagulation with low molecular weight heparin was initiated given persistent atrial fibrillation/flutter and prior cardioversion attempts.

Antithyroid therapy

Propylthiouracil (PTU) was loaded at a dose of 500 mg on Day 2, followed by a maintenance dose of 200 mg three times daily from Day 7. PTU was gradually weaned until Day 14.

Outcome and follow-up

Serial thyroid biochemistry demonstrated progressive improvement, with normalisation of serum T3 concentrations following treatment. Lugol's iodine was discontinued on Day 6 and cholestyramine on Day 8. PTU was gradually weaned in response to improving thyroid function tests.

Repeat echocardiography on Day 5 demonstrated improvement in left ventricular systolic function, with ejection fraction increasing from 15-20% to approximately 30%. Functional tricuspid regurgitation and pulmonary hypertension parameters also improved on interval imaging, further supporting a reversible tachycardia-mediated mechanism [5].

Despite these improvements, the patient remained in persistent atrial fibrillation/flutter with recurrent episodes of fluid overload, requiring ongoing diuretic therapy and careful titration of rate-control agents. The patient remained under joint endocrinology and cardiology management for continued optimisation of thyroid disease, ventricular rate control and heart failure therapy.

Long-term management is focused on strict ventricular rate control and endocrine optimisation. The patient was cardioverted prior to a transoesophageal echocardiography as it was deemed necessary at the time.

## Discussion

This case illustrates a severe and complex presentation of thyroid storm, characterised by refractory atrial flutter, haemodynamic instability and tachycardia-induced dilated cardiomyopathy. The Burch-Wartofsky score of 95 at presentation reflects extreme thyrotoxic decompensation, and the clinical picture was further complicated by the degree of cardiac dysfunction observed on echocardiography. Older age (≥60 years), central nervous system dysfunction, non-use of antithyroid drugs and beta-blockade, and requirement for mechanical ventilation have all been independently associated with higher in-hospital mortality in thyroid storm [2].

Atrial arrhythmias are a well-established cardiovascular manifestation of thyrotoxicosis, driven by thyroid hormone-mediated shortening of the atrial refractory period, enhanced automaticity and increased adrenergic sensitivity; subclinical hyperthyroidism alone is associated with a three-fold increased risk of developing atrial fibrillation, underscoring the potency of thyroid hormone effects on atrial electrophysiology [4,8]. At the molecular level, triiodothyronine (T3) downregulates atrial L-type calcium channel expression, abbreviating action potential duration and creating a substrate for atrial arrhythmias [9]. Atrial flutter, whilst less common than atrial fibrillation in this context, may present with a rapid ventricular response that is particularly resistant to pharmacological cardioversion. In our patient, the arrhythmia proved refractory to adenosine, three direct-current cardioversions and multiple antiarrhythmic agents, underscoring the importance of treating the underlying endocrine cause alongside cardiac stabilisation [1].

Tachycardia-induced cardiomyopathy is a recognised but potentially underappreciated complication of sustained supraventricular tachyarrhythmia. The pathophysiology involves energy depletion at the myocardial level, calcium dysregulation and progressive chamber dilatation, which may mimic primary dilated cardiomyopathy. In a series of 24 patients with tachycardia-induced cardiomyopathy, mean left ventricular ejection fraction (LVEF) at presentation was 0.26±0.09, with improvement or normalisation of LVEF in all patients within six months of rate or rhythm control; however, recurrent tachycardia caused rapid decline in ventricular function, and three patients died suddenly [5]. Echocardiographic predictors of tachycardia-induced cardiomyopathy include normal indexed left ventricular end-diastolic volume (OR 5.2; 95% CI 2.2-13.3), fractional shortening <30% (OR 3.5; 95% CI 1.4-9.2) and left atrial volume index >40 mL/m^2^ (OR 3.4; 95% CI 1.6-7.3) [7]. Importantly, even after arrhythmia cure, diffuse left ventricular fibrosis has been demonstrated on cardiac MRI in tachycardia-mediated cardiomyopathy patients, suggesting that recovery may be incomplete despite normalisation of ejection fraction [10]. In this case, the rapid and partial improvement in ejection fraction following ventricular rate control is consistent with a tachycardia-mediated aetiology and supports the potential for further recovery with ongoing management.

The decision to discontinue amiodarone warrants specific comment. Amiodarone is one of the most effective antiarrhythmic agents for supraventricular tachycardia; however, its use in thyrotoxicosis is problematic. Amiodarone contains approximately 37% iodine by weight and can precipitate or worsen thyrotoxicosis through multiple mechanisms, including both iodine-induced thyroid dysfunction (Jod-Basedow phenomenon) and direct cytotoxic effects on thyroid tissue [1]. Moreover, amiodarone inhibits peripheral T4-to-T3 conversion, complicating interpretation of thyroid function tests. Its prolonged tissue half-life (40-55 days) means that adverse thyroid effects may persist long after discontinuation. Following multidisciplinary review, the decision to discontinue amiodarone and transition to a rate-control strategy with bisoprolol and digoxin was appropriate in this context.

Lugol's iodine, used as part of thyroid storm management, carries its own risks in a patient with Graves' disease, as prolonged administration may paradoxically escape the Wolff-Chaikoff block and exacerbate thyrotoxicosis; timely discontinuation, as occurred in this case, is therefore important. Cholestyramine acts as an ion exchange resin that interrupts the enterohepatic recirculation of thyroid hormones and has been used as an adjunct in severe thyroid storm, with early discontinuation in this case following biochemical improvement [1].

The bilateral proptosis observed clinically, in conjunction with the pattern of biochemical thyrotoxicosis, is consistent with an underlying diagnosis of Graves' disease. This is further supported by a markedly elevated TRAb and neck ultrasound appearances demonstrating diffuse thyroid enlargement, hypoechoic coarsened parenchyma and markedly increased vascularity, sonographic features characteristic of Graves' disease. Further outpatient evaluation will be required to guide definitive management, including consideration of radioiodine therapy, thyroidectomy or long-term antithyroid drug therapy [1].

More broadly, this case underscores the spectrum of cardiovascular decompensation that can accompany thyroid storm. Cardiogenic shock, while a less common cardiovascular complication than arrhythmia or heart failure, is likewise associated with substantial in-hospital mortality and healthcare burden, reinforcing the need for early recognition and aggressive multidisciplinary management of cardiovascular involvement in thyroid storm more generally [11].

Beyond the cardiovascular system, thyroid storm frequently produces broader metabolic derangement, including hyperglycaemia, elevated lactate, leukocytosis in the absence of infection, and mild hypercalcaemia; hepatic transaminases and alkaline phosphatase may also be elevated. When thyroid storm coexists with decompensated heart failure, hepatic dysfunction may reflect a combination of direct thyrotoxic effects and congestive hepatopathy; this pattern has been described in a reported case of thyroid storm complicated by heart failure with reduced ejection fraction, in which markedly elevated transaminases and bilirubin accompanied severe biventricular dysfunction [1].

**Figure 4:**
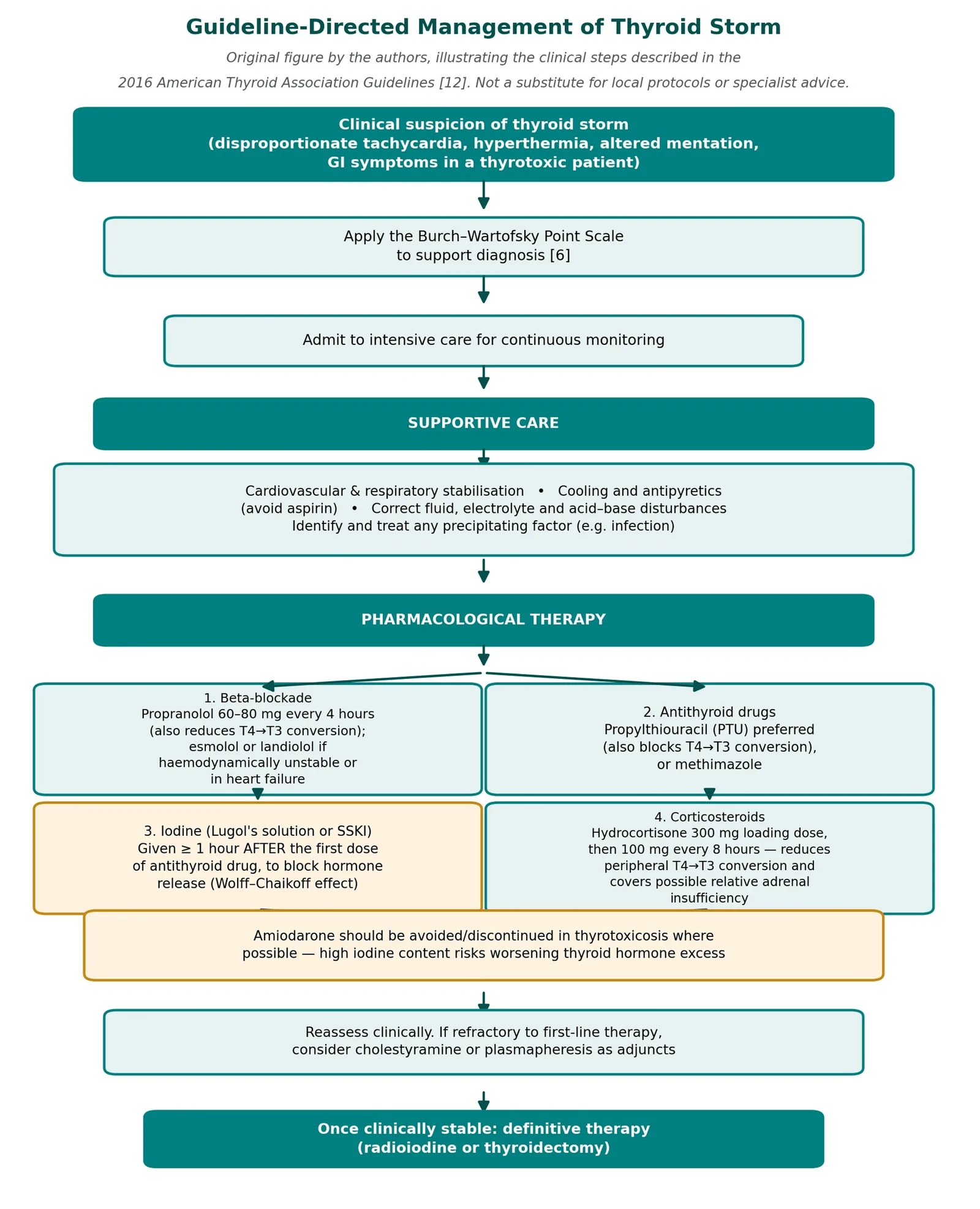
Guideline-directed management algorithm for thyroid storm. GI: gastrointestinal; DKA: diabetic ketoacidosis; JTA: Japan Thyroid Association; JES: Japan Endocrine Society; KSSKI: saturated solution of potassium iodide; T3: triiodothyronine; T4: thyroxine Source: The figure was created by the authors using Claude AI (Anthropic PBC, San Francisco, CA, USA) based on the clinical recommendations described in the 2016 American Thyroid Association Guidelines [12]. The content and scientific accuracy of the figure were reviewed and verified by Favour O. Balogun.

To support translation of these principles into acute practice, Figure 4 summarises a guideline-directed management algorithm for thyroid storm [12], adapted for the ED/ICU setting.

## Conclusions

This case demonstrates the diagnostic and therapeutic complexity of thyroid storm presenting with refractory atrial arrhythmia and tachycardia-induced dilated cardiomyopathy. Early identification of thyroid storm as the underlying diagnosis is critical, as management must simultaneously address the endocrine emergency, the refractory arrhythmia, and the resultant cardiac dysfunction. As discussed above, the cardiovascular complications of thyroid storm are associated with significantly increased morbidity and mortality, reinforcing the need for prompt and targeted intervention.

The partial recovery in left ventricular function observed in this patient following rate control and endocrine treatment is encouraging and consistent with a reversible tachycardia-mediated mechanism. However, as outlined above, the possibility of residual myocardial fibrosis despite apparent functional recovery warrants long-term surveillance. Ongoing multidisciplinary input from endocrinology, cardiology and critical care remains fundamental to optimising outcomes in such complex presentations, and clinicians should maintain a high index of suspicion for thyroid storm in any patient presenting with unexplained refractory tachyarrhythmia and multisystem decompensation.
